# Clinical Outcomes and Risk Factors for Death following Carbapenem-Resistant Klebsiella pneumoniae Infection in Solid Organ Transplant Recipients

**DOI:** 10.1128/spectrum.04755-22

**Published:** 2022-12-14

**Authors:** Jingli Lu, Ailing Zhang, Lu Han, Zhen Guo, Wenjing Cui, Yuhua Jiang, Haiyang Meng

**Affiliations:** a Department of Pharmacy, First Affiliated Hospital of Zhengzhou University, Zhengzhou, China; b Henan Key Laboratory of Precision Clinical Pharmacy, Zhengzhou University, Zhengzhou, China; c Second People’s Hospital of Zhengzhou, Zhengzhou, China; d First People’s Hospital of Xiangcheng, Xiangcheng, China; e Xiangcheng Hospital of Chinese Medicine, Xiangcheng, China; f Affiliated Hospital of Huanghe Science and Technology University, Zhengzhou, China; University of Mississippi Medical Center

**Keywords:** carbapenem-resistant *Klebsiella pneumoniae*, organ transplant, septic shock, allograft failure, appropriate targeted therapy

## Abstract

Infections caused by carbapenem-resistant Klebsiella pneumoniae (CRKP) are associated with significant morbidity and mortality. Among solid organ transplant recipients (SOTRs), clinical outcomes and risk factors for death following such infections remain not well documented. A single-center retrospective study was performed. All SOTRs with a CRKP infection at the First Affiliated Hospital of Zhengzhou University between 1 January 2018 and 31 December 2021 were included. Multivariable Cox regression was performed to determine risk factors for death following CRKP infection. We identified 94 SOTRs with CRKP infection, with a median age of 50 years old. CRKP infections resulted in 38.3% of overall 30-day mortality. On multivariable analysis, independent risk factors for death following CRKP infection included older age (hazard ratio [HR], 1.044; 95% confidence interval [CI], 1.007 to 1.083; *P* = 0.02), allograft failure (HR, 3.962; 95% CI, 1.628 to 9.644; *P* = 0.002), and septic shock (HR, 8.512; 95% CI, 3.294 to 21.998; *P* < 0.001). Receiving appropriate targeted therapy was associated with a reduced hazard of death (HR, 0.245; 95% CI, 0.111 to 0.543; *P* = 0.001). Our study characterized the clinical features and mortality in SOTRs with CRKP infection. The protective effects of appropriate targeted therapy highlight the importance of assessing how antibiotic choices affect the clinical outcomes among SOTRs.

**IMPORTANCE** Carbapenem-resistant Klebsiella pneumoniae (CRKP) infections are increasingly identified in solid organ transplant recipients (SOTRs), but data on the clinical outcomes and risk factors for death following such infections remain limited. Here, we reported CRKP infection was associated with 38.3% of overall 30-day mortality in SOTRs. Independent risk factors for death after CRKP infection included older age, allograft failure, and septic shock. Appropriate targeted therapy was important for alleviating the impact of CRKP infections on these SOTRs.

## INTRODUCTION

Carbapenem-resistant *Enterobacteriaceae* (CRE) are a public health concern; infections caused by these organisms are associated with high mortality and limited treatment options (1). Among them, the carbapenem-resistant Klebsiella pneumoniae (CRKP) is of particular concern because it is the most common *Enterobacterales* species that is resistant to carbapenems (2). In China, K. pneumoniae strains account for up to 60% of CRE infections (3). According to the China Antimicrobial Surveillance Network (CHINET, http://www.chinets.com/), the prevalence of carbapenem resistance in clinical K. pneumoniae increased by nearly 8-fold in the past 15 years, from 2.9% in 2005 to 24.2% in 2020 (based on meropenem resistance) (4). Although global CRKP epidemics vary regionally, CRKP infection has emerged as a major threat in hospitals worldwide (5–7).

CRKP infections occur more frequently in solid organ transplant recipients (SOTRs) (8, 9). SOTRs are exposed to broad-spectrum antibiotics, surgical interventions, and the use of immunosuppressants, which drive an increased risk of infection (8–10). In particular, clinical outcomes following CRKP infection in SOTRs are poor, with a mortality rate five times greater than those without CRKP infection (11). CRKP infection has reported a 71% overall mortality among liver transplant recipients (12). In 43 kidney transplant recipients with early CRKP infection, the rate of infection-related mortality was 37.2% (13). Another study of kidney transplant recipients found that CRKP bacteriuria was associated with increased overall mortality of 30% but not graft failure (14). It reveals that the rates of death vary depending on the type of organ transplant studied and infection types. As a result, it is crucial to identify risk factors for clinical outcomes following CRKP infection among SOTRs.

However, previous studies are limited by the small sample size and types of organ transplants. In this study, we sought to describe (i) the clinical characteristics, (ii) the impacts of different antibiotics on clinical outcomes, and (iii) the risk factors for mortality following CRKP infection, using a cohort of solid organ transplant types.

## RESULTS

### Study population and clinical characteristics.

Baseline characteristics of the cohort stratified by organ type are listed in Table 1. Of 94 unique individuals with CRKP infection after organ transplant, 53 received a liver transplant, 21 received a kidney transplant, and 20 received a lung transplant. The median age was 50 years (interquartile range [IQR], 42 to 57); 83.0% were male. The most commonly reported comorbidities included hypertension (29.8%) and diabetes (14.9%). In the 3 months prior to the CRKP infection, 72 (76.6%) had been admitted to the intensive care unit (ICU), and 78 (83.0%) had received carbapenem therapy. Median time from admission to the diagnosis of CRKP infection was 19 days (6 to 36 days). A total of 73.4% of patients developed CRKP infections within 3 months post-transplant, and 53.2% of patients received induction immunosuppression within 2 months before the CRKP infection. More than 70% of patients received corticosteroids, mycophenolate, and tacrolimus as maintenance immunosuppression. Lung recipients were more likely to have a longer stay in the ICU and longer duration of mechanical ventilation. Kidney recipients more often have diabetes and are more often hospitalized due to infection.

**TABLE 1:** Characteristics of solid organ transplant recipients with CRKP infection^*a*^

Characteristics	All patients (*n* = 94)	Liver (*n* = 53)	Kidney (*n* = 21)	Lung (*n* = 20)	*P* value^*b*^
Demographics					
Age (yr)	50 (42 to 57)	49 (43 to 55)	49 (39 to 56)	54 (37 to 62)	0.339
Sex, male	78 (83.0%)	49 (92.5%)	12 (57.1%)	17 (85.0%)	0.001
Comorbidities at the time of CRKP infection					
Diabetes mellitus	14 (14.9%)	10 (18.9%)	3 (14.3%)	1 (5.0%)	0.393
Hypertension	28 (29.8%)	12 (22.6%)	15 (71.4%)	1 (5.0%)	<0.001
aCCI	3 (3 to 5)	5 (3 to 6)	3 (2 to 3)	3 (1 to 3)	<0.001
Reason for admission					
Transplant operation	64 (68.1%)	42 (79.2%)	6 (28.6%)	16 (90.0%)	0.001
Infection	18 (19.1%）	5 (9.4%)	10 (47.6%)	3 (15.0%)	
Allograft function declined	3 (3.2%)	2 (3.8%)	1 (4.8%)	0 (0)	
Other systemic symptoms	9 (9.6%)	4 (7.5%)	4 (19.0%)	1 (5.0%)	
Healthcare and antibiotic exposures prior to CRKP infection					
Carbapenem exposure within 3 mo	78 (83.0%)	45 (84.9%)	14 (66.7%)	19 (95.0%)	0.058
Surgery within 30 days	65 (69.1%)	41 (77.4%)	7 (33.3%)	17 (85.0%)	<0.001
ICU admission within 3 mo	72 (76.6%)	43 (81.1%)	9 (42.9%)	20 (100%)	<0.001
Transplant characteristics					
Time from admission to CRKP infection (days)	19 (6 to 36)	22 (11 to 43)	10 (2 to 23)	19 (6 to 48)	0.012
Infection time <3 mo post-transplant	69 (73.4%)	46 (86.8%)	6 (28.6%)	17 (85.0%)	<0.001
Use of induction immunosuppression within 2 mo before the CRKP infection					
Basiliximab/ATG	50 (53.2%)	43 (81.1%)	3 (14.3%)	4 (20.0%)	<0.001
Use of immunosuppression during the time of the CRKP infection					
Corticosteroids	70 (74.5%)	34 (64.2%)	17 (81.0%)	19 (95.0%)	0.019
Mycophenolate	72 (76.6%)	50 (94.3%)	14 (66.7%)	8 (40.0%)	<0.001
Tacrolimus	82 (87.2%)	48 (90.6%)	17 (81.0%)	17 (85.0%)	0.497
Sirolimus	14 (14.9%)	14 (26.4%)	0 (0)	0 (0)	0.001
Cyclosporine	10 (10.6%)	2 (3.8%)	4 (19.0%)	4 (20.0%)	0.027
Location at the time of CRKP infection					
Medical ward, non-ICU	5 (5.3%)	4 (7.5%)	1 (4.8%)	0 (0)	0.104
Surgical ward, non-ICU	38 (40.4%)	25 (47.2%)	9 (42.9%)	4 (20.0%)	
ICU	51 (54.3%)	24 (45.3%)	11 (52.4%)	16 (80.0%)	
Severity of illness and complications during the CRKP infection					
ICU admission	61 (64.9%)	32 (60.4%)	12 (57,1%)	17 (85.0%)	0.110
Length of ICU (days)	2 (0 to 9)	2 (0 to 5)	2 (0 to 6.5)	9.5 (3.25 to 17)	0.002
Mechanical ventilation	47 (50%)	24 (45.3%)	8 (38.1%)	15 (75.0%)	0.036
Duration of mechanical ventilation (days)	10 (0 to 4)	0 (0 to 2)	0 (0 to 3)	7 (0 to 10)	0.003
Respiratory failure^*c*^	29 (30.9%)	11 (20.8%)	9 (42.9%)	9 (45.0%)	0.061
Requirement of CRRT	22 (23.4%)	11 (20.8%)	6 (28.6%)	5 (25.0%)	0.756
Allograft failure^*d*^	32 (34.0%)	16 (30.2%)	5 (23.8%)	11 (55.0%)	0.067
Septic shock	26 (27.7%)	14 (26.4%)	7 (33.3%)	5 (25.0%)	0.817
Laboratory variables at the time of CRKP infection					
Absolute neutrophil count (10^9^ cells/liter)	6.3 (3.8 to 10.0)	5.2 (3.2 to 8.2)	5.5 (3.7 to 8.3)	11.8 (6.5 to 15.7)	<0.001
Absolute white blood cell count (10^9^ cells/liter)	7.2 (4.7 to 11.0)	6.0 (4.0 to 9.1)	7.1 (4.6 to 9.9)	11.9 (7.4 to 16.4)	0.001
Creatinine (mg/dL)	90.0 (61.8 to 158.0)	89.1 (61.5 to 134)	159.0 (114.7 to 390.0)	66.0 (39.8 to 84.8)	<0.001
Alkaline phosphatase (U/liter)	106.5 (67.0 to 162.0)	132.0 (80.0 to 185.0)	113 (55.0 to 165.1)	68 (51.75 to 114.5)	0.002
Total bilirubin (μmol/L)	19.5 (10.0 to 67.7)	47.2 (21.1 to 166.3)	7.9 (5.5 to 12.8)	14.4 (9.7 to 19.3)	<0.001
Albumin (g/liter)	37.1 (32.8 to 42.4)	36.5 (33.1 to 40.9)	34.6 (27.2 to 38.4)	42.1 (38.4 to 46.5)	<0.001

a The data are *n* (%) or median (interquartile range). aCCI, age-adjusted Charlson comorbidity index; anti-thymocyte globulin; CRKP, carbapenem-resistant K. pneumoniae; CRRT, continuous renal replacement therapy; ICU, intensive care unit.

b *P* value comparing liver, kidney, and lung transplant, as well as distributions, where applicable.

c A total of 29 patients experienced respiratory failure; 25 of them received mechanical ventilation.

d Presence of allograft failure included graft failure prior to the CRKP infection (*n* = 19) and new-onset graft failure (*n* = 13).

### Characteristics of CRKP infections.

**FIG 1:**
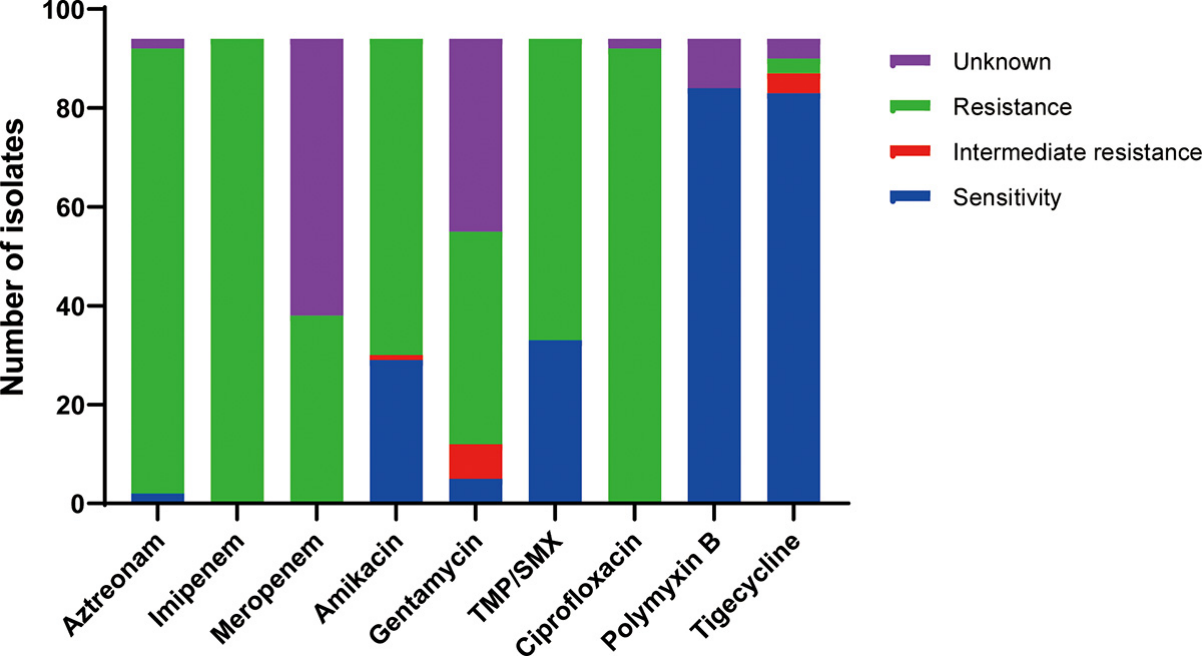
*In vitro* antibiotic susceptibility. TMP/SMX, trimethoprim/sulfamethoxazole.

Lung infections were the most common type occurring post-transplant (*n* = 60, 63.8%), followed by bloodstream infections (BSIs, *n* = 27, 28.7%), intra-abdominal infections (*n* = 9, 9.6%), and surgical site infections (*n* = 8, 8.5%). Five patients (5.3%) had genitourinary tract infections, five (5.3%) had hepatobiliary infections, and two (2.1%) had skin and soft tissue infections (Table 2). There were 15 patients of 94 (16%) who had infections in more than one site (Table 2). All isolates were resistant to imipenem, meropenem, and ciprofloxacin. The rate of susceptibility to aztreonam, amikacin, gentamicin, and TMP-SMX were, respectively, 2.2%, 31.9%, 21.8%, and 35.1%. Susceptibility to tigecycline was at relatively high levels (96.7%, 87 of 90). All isolates were susceptible to polymyxin B (Fig. 1).

**TABLE 2:** Characteristics of CRKP infections^*a*^

Characteristics	All patients (*n* = 94)	Liver (*n* = 53)	Kidney (*n* = 21)	Lung (*n* = 20)	*P* value^*b*^
Infection types					
Lung infection	60 (63.8%)	33 (62.3%)	9 (42.9%)	18 (90.0%)	0.006
Intra-abdominal infection	9 (9.6%)	9 (17.0%)	0 (0)	0 (0)	0.014
BSI	27 (28.7%)	17 (32.1%)	7 (33.3%)	3 (15.0%)	0.335
Genitourinary tract infection	5 (5.3%)	0 (0)	5 (23.8%)	0 (0)	0.001
Skin and soft tissue infection	2 (2.1%)	1 (1.9%)	0 (0)	1 (5.0%)	0.430
Hepatobiliary infection	5 (5.3%)	5 (9.4%)	0 (0)	0 (0)	0.226
Surgical site infection	8 (8.5%)	4 (7.5%)	3 (14.3%)	1 (5.0%)	0.597
No. of infection sites					
1	79 (84.0%)	44 (83.0%)	18 (85.7%)	17 (85.0%)	0.962
2	11 (11.7%)	5 (9.4%)	3 (14.3%)	3 (15.0%)	
3	2 (2.1%)	2 (3.8%)	0 (0)	0 (0)	
4	2 (2.1%)	2 (3.8%)	0 (0)	0 (0)	

a The data are *n* (%). BSI, bloodstream infection; CRKP, carbapenem-resistant K. pneumoniae.

b *P* value comparing liver, kidney, and lung transplant, as well as distributions, where applicable.

### Treatment and outcomes of CRKP infections.

Appropriate empirical therapy and targeted therapy were given to 30.9% and 71.3% of patients with CRKP infections, respectively; 26.6% received at least one active antibacterial agent within 24 h after infection; and 13.8% received two or three active antibiotics (*n* = 13). Tigecycline and carbapenems were the most common antimicrobials, with both used in 38 cases. Twelve patients (12.8%) received an antibiotic regimen containing polymyxin B, 27 (28.7%) ceftazidime-avibactam (CAZ-AVI), and 28 (29.8%) other β-lactam antibiotics. Mortality rates at 14 days, 30 days, and 6 months after initial CRKP infections were 28.7%, 38.3%, and 46.8%, respectively (Table 3).

**TABLE 3:** Management of CRKP infection and outcomes^*a*^

Management	All patients (*n* = 94)	Liver (*n* = 53)	Kidney (*n* = 21)	Lung (*n* = 20)	*P* value^*b*^
Effective antibiotic received					
Appropriate empirical therapy	29 (30.9%)	15 (28.3%)	4 (19.0%)	10 (50.0%)	0.084
Appropriate targeted therapy^*c*^	67 (71.3%)	38 (71.7%)	12 (57.1%)	17 (85.0%)	0.140
Effective antibiotics within 24 hours	25 (26.6%)	13 (24.5%)	3 (14.3%)	9 (45.0%)	0.074
Targeted antibiotic therapy					
Tigecycline	38 (40.4%)	23 (43.4%)	8 (38.1%)	7 (35.0%)	0.812
Polymyxin B	12 (12.8%)	1 (1.9%)	1 (4.8%)	10 (50.0%)	<0.001
CAZ-AVI	27 (28.7%)	17 (32.1%)	3 (14.3%)	7 (35.0%)	0.264
Carbapenems	38 (40.4%)	22 (41.5%)	10 (47.6%)	6 (30.0%)	0.512
Other β-lactam antibiotics^*d*^	28 (29.8%)	18 (34.0%)	5 (23.8%)	5 (25.0%)	0.620
Aminoglycosides	6 (6.4%)	2 (3.8%)	1 (4.8%)	3 (15.0%)	0.212
Fluoroquinolones	7 (7.4%)	4 (7.5%)	1 (4.8%)	2 (10%)	0.880
TMP/SMX	5 (5.3%)	0 (0)	3 (14.3%)	2 (10%)	0.014
Fosfomycin	1 (1.1%)	0 (0)	0 (0)	1 (5.0%)	0.213
14-day mortality	27 (28.7%)	15 (28.3%)	7 (33.3%)	5 (25.0%)	0.864
30-day mortality	36 (38.3%)	19 (35.8%)	10 (47.6%)	7 (35.0%)	0.626
6-mo mortality	44 (46.8%)	22 (41.5%)	11 (52.4%)	11 (55.0%)	0.522

a The data are n (%). CAZ-AVI, ceftazidime-avibactam; CRKP, carbapenem-resistant K. pneumoniae; TMP/SMX, trimethoprim-sulfamethoxazole.

b *P* value comparing liver, kidney, and lung transplant, as well as distributions, where applicable.

c A total of 27 patients did not receive appropriate targeted therapy, 7 died prior to the availability of susceptibility results, and 1 patient was discharged alive prior to the availability of susceptibility results.

d Other β-lactam antibiotics: aztreonam (*n* = 2); cefoperazone/sulbactam (*n* = 19); mezlocillin/sulbactam (*n* = 3); piperacillin-tazobactam (*n* = 3); and ceftizoxime (*n* = 1).

**FIG 2:**
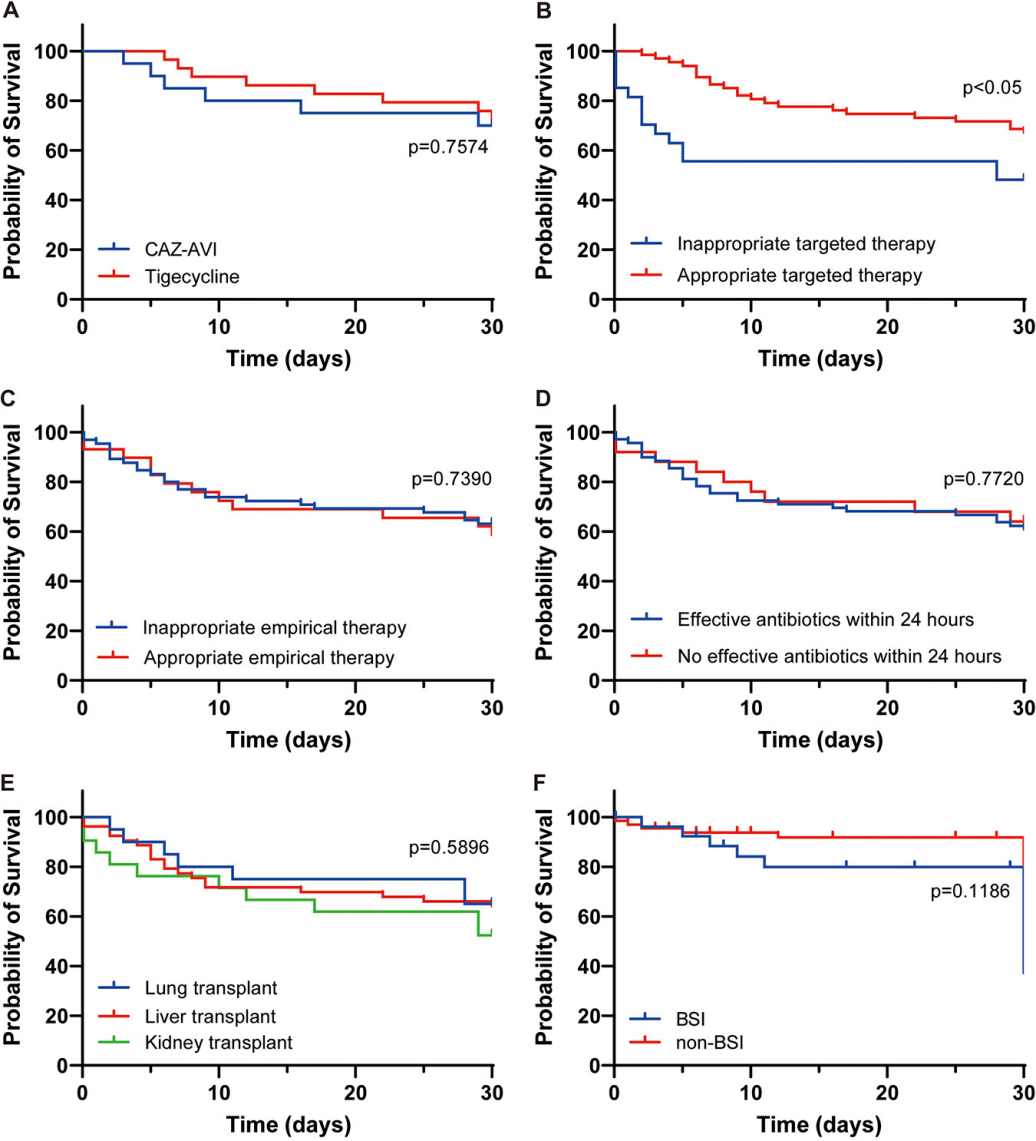
Kaplan-Meier curves for all-cause mortality. (A) Survival for solid organ transplant recipients (SOTRs) with carbapenem-resistant K. pneumoniae (CRKP) infection within 30 days by active antibacterial agent. (B) Survival for SOTRs with CRKP infection within 30 days by targeted therapy. (C) Survival for SOTRs with CRKP infection within 30 days by empirical therapy. (D) Survival for SOTRs with CRKP infection within 30 days by effective antibiotics within 24 h. (E) Survival for SOTRs with CRKP infection within 30 days by type of organ transplant. (F) Survival for SOTRs with CRKP infection within 30 days by infection site. BSI, bloodstream infection; CAZ-AVI, ceftazidime-avibactam.

With regard to three active antibacterial agents, all-cause mortality at day 30 was 30.0% (6 of 20) for the CAZ-AVI monotherapy, 66.7% (4 of 6) for the polymyxin B monotherapy, and 27.6% (8 of 29) for the tigecycline monotherapy (Table 4). Regarding all-cause mortality in patients receiving CAZ-AVI monotherapy and tigecycline monotherapy, survival curves crossed, and no differences were observed (Fig. 2A). The numbers in other therapeutic regimens were too low for meaningful analysis.

**TABLE 4:** Impacts of active antimicrobial regimens on the mortality of patients with CRKP infection in SOTRs^*a*^

Antimicrobial regimens	All patients	Infection sites^*b*^	
		Respiratory infection	BSI
CAZ-AVI	6/20 (30.0%)	4/15 (26.7%)	2/5 (40.0%)
CAZ-AVI + polymyxin B	0/2 (0)	0/1 (0)	0/1 (0)
CAZ-AVI + tigecycline	2/5 (40.0%)	0/1 (0)	0/1 (0)
Polymyxin B	4/6 (66.7%)	3/5 (60.0%)	1/2 (50.0%)
Polymyxin B + tigecycline	1/4 (25.0%)	1/4 (25.0%)	0/0 (0)
Tigecycline	8/29 (27.6%)	6/17 (35.3%)	2/8 (25.0%)

a The data are *n*/*N* (%), where *N* is the number of patients receiving respective antimicrobial regimens, and *n* is the number of patients who died within 30 days. BSI, bloodstream infection; CAZ-AVI, ceftazidime-avibactam; CRKP, carbapenem-resistant K. pneumoniae; SOTR, solid organ transplant recipient.

b Other infection sites have been omitted due to limited numbers.

All-cause 30-day mortality was lower in patients who received an appropriate targeted therapy than those who received inappropriate targeted therapy (*P* < 0.05) (Fig. 2B). No significant difference was found in 30-day mortality stratified by the appropriate empirical therapy (Fig. 2C), effective therapy within 24 h (Fig. 2D), types of organ transplant (Fig. 2E), and infection sites (BSI versus non-BSI) (Fig. 2F).

### Risk factors for death following CRKP infection.

In the multivariable analysis, the independent risk factors for death within 30 days included older age (hazard ratio [HR], 1.044; 95% confidence interval [CI], 1.007 to 1.083; *P* = 0.02), allograft failure (HR, 3.962; 95% CI, 1.628 to 9.644; *P* = 0.002), and septic shock (HR, 8.512; 95% CI, 3.294 to 21.998; *P* < 0.001). Conversely, receiving appropriate targeted therapy was associated with a reduced hazard of death (HR, 0.245; 95% CI, 0.111 to 0.543; *P* = 0.001) (Table 5).

**TABLE 5:** Multivariable Cox regression of risk factors for death following CRKP infection^*a*^

Risk factors	HR	95% CI	*P* value
All patients			
Older age	1.044	1.007 to 1.083	0.02
Allograft failure	3.962	1.628 to 9.644	0.002
Septic shock	8.512	3.294 to 21.998	<0.001
Appropriate targeted therapy	0.245	0.111 to 0.543	0.001
Liver transplant recipients^*b*^			
Allograft failure	9.198	3.026 to 27.954	<0.001
Patients with lung infection^*b*^			
Older age	1.117	1.030 to 1.212	0.008
Allograft failure	6.660	2.026 to 21.892	0.002
Septic shock	4.576	1.608 to 13.024	0.004
Infection time less than 3 mo post-transplant	0.171	0.062 to 0.472	0.001

a CI, confidence interval; CRKP, carbapenem-resistant K. pneumoniae; HR, hazard ratio.

b The values are for lung and kidney transplant recipients; other infection sites have been omitted due to insufficient numbers for analysis. The relevant analysis for other subgroups was not implemented because of the limited sample size.

We did a subgroup analysis. Among liver transplant recipients, there was a significant association between allograft failure and the hazard of mortality (HR, 9.198; 95% CI, 3.026 to 27.954; *P* < 0.001). After stratifying infection sites, in SOTRs with lung infection, older age (HR, 1.117; 95% CI, 1.03 to 1.212; *P* = 0.008), allograft failure (HR, 6.66; 95% CI, 2.026 to 21.892; *P* = 0.002), and septic shock (HR, 4.576; 95% CI, 1.608 to 13.024; *P* = 0.004) were associated with greater hazard of mortality, whereas time to infection after transplant less than 3 months was associated reduced hazard of death (HR, 0.171; 95% CI, 0.062 to 0.472; *P* = 0.001) (Table 5).

## DISCUSSION

In our retrospective cohort study, 94 CRKP cases were reported across three types of solid organ transplant populations, resulting in 38.3% overall 30-day mortality. This study represents the largest cohort of SOTRs with CRKP infection documented to date. Previous studies have found substantially higher rates of death (12, 15, 16), reaching up to 71% in liver transplant (12). The observed mortality difference might be attributed to the variation by types of organ transplant and infection sites, as well as the duration of follow-up and sample size (12–17). In addition, during the study period, the failure to access novel anti-CRKP antibiotics, such as CAZ-AVI, could have led to high mortality (12, 16).

In our multivariate analysis, the most predictive factor was septic shock, with an 8-fold increased risk of 30-day mortality. Septic shock has been shown to be a risk factor for mortality from CRKP infection in nontransplant patients (18–20) and carbapenem-resistant Gram-negative bacteria among abdominal SOTRs (21). Septic shock is a life-threatening disorder defined as sepsis with hypotension and perfusion abnormalities, with in-hospital mortality rates approaching 30 to 50% (22). SOTRs with sepsis had a significant relative reduction in mortality outcomes compared with nontransplant patients, because the immunosuppression used in SOTRs could modulate dysregulated systemic inflammatory and immune response to microbial invasion (23). However, severe sepsis remains among the main causes of death in SOTRs (24). Thus, septic shock caused by CRKP infection may accelerate death in SOTRs.

In addition to septic shock, we identified other risk factors for death following CRKP infection that was important to the SOTR population. First, we found that older age was associated with an increased hazard of death in all SOTRs, which has been confirmed in nontransplanted patients with CRKP respiratory infection (4). Consistent with this, in our subgroup analysis of infection sites, older age was an attributable factor in death due to CRKP lung infection. These results underscored the importance of respiratory defenses in CRKP infection. Second, allograft failure was an independent risk factor for death. Indeed, since septic shock is accompanied by multiorgan failure, allograft dysfunctions may become more apparent during the sepsis episode. The presence of allograft failure likely reflects that these patients are more severely ill and require more hospital procedures, such as mechanical ventilation and CRRT, which were identified as potential risk factors in our univariable analysis.

Previous studies have found that appropriate empirical antibiotic therapy was an important protective factor in patients with CRKP infection (25, 26). Appropriate antibiotic therapy within 24 h results in 30% reduction in 30-day mortality rates (26). In our study, effective antibiotic therapy within 24 or 72 h did not show a protective effect on the prognosis of patients with CRKP infection. A similar result was observed in nontransplant patients from previous reports in China (4). Nevertheless, we indeed demonstrated appropriate targeted antibiotic therapy was associated with a reduced hazard of death in SOTRs, but we failed to identify specific antibiotics to use as a protective factor.

Regarding the active antibiotics used, treatments for CRKP infection were primarily based on tigecycline (40.4%). A similar antibiotic pattern was reported in a recent study that surveyed the clinical management of CRKP in mainland China; it reported that tigecycline was the most prescribed antibiotic (27). Our study included 64% of lung infection and 29% of BSI, which are not recommended indicators for tigecycline. It reinforced concerns that tigecycline may be associated with increased mortality, which has been confirmed in nontransplant patients (4). In contrast, CAZ-AVI—superior to polymyxins and tigecycline—is considered to be the preferred treatment for patients with severe CRKP infection (20, 28, 29). Thus, treatment of an antibiotic regimen containing CAZ-AVI was an independent protective factor for 30-day mortality in kidney transplant (30). However, only a subset of patients received CAZ-AVI as empirical (4.3%) and targeted therapy (28.7%) in our study. Unexpectedly, in multivariate analysis, we failed to observe the association between antibiotics and mortality; we found no difference in 30-day mortality among patients who received CAZ-AVI monotherapy and tigecycline monotherapy. This discrepancy may stem from the difference in disease severity. In our study, patients treated with tigecycline experienced less severe infection, whereas CAZ-AVI as salvage therapy was used in patients with higher disease severity. This hypothesis can be supported by the proportion of ICU admission (66.7%) and mechanical ventilation requirement (55.6%) in the CAZ-AVI group, compared with 55.2 and 31.0%, respectively, in the tigecycline group. Also, the small size of the sample population should be considered. Therefore, it is necessary to further evaluate the efficacy of active antibiotics in the treatment of CRKP infection in the SOTR population.

Interestingly, we found that the onset of infection within first 3 months after transplant was associated with reduced hazard of death in SOTRs with lung infection. This finding appears to be counterintuitive, given that the majority of infections occurred during the early post-transplant phase (31, 32), and these patients have had poorer survival outcomes due to their intensive immunosuppressive regimens and recent surgical procedures. Our results may be explained in several ways. First, nearly one-third of the patients with CRKP infection in our study developed septic shock. Patients with septic shock often present with massive proinflammatory cytokine-driven hyperinflammatory at the early phase; hence, intense levels of immunosuppression during the early post-transplant phase could limit excessive inflammation and protect these patients from entering an overwhelming hyperinflammatory phase, which substantially prevents disease progression and improves sepsis mortality (33, 34). Another possible explanation is the fact that allograft function declines progressively over time (35, 36), which is one important independent factor for death after infection (37). We found that 28.6% of infections (4 of 14) occurred in the 4- to 12-year period after transplant; therefore, it is possible that chronic allograft damage associated with the late post-transplant period could be attributed to the high mortality rate (37). This hypothesis is supported by our finding that we observed a trend toward high mortality in kidney transplant (47.6%) compared with lung transplant (35.0%) or liver transplant (35.8%). This is likely attributed to the longer time from kidney transplant to CRKP infection, with a median time of 1,518 days.

With regard to types of infection, lung (64%) is the most common source of CRKP infection, followed by BSI (29%). BSI likely accounted for the high overall mortality observed (48.1%), although no significant difference was found. Previous studies also have demonstrated a greater severity of illness and death in nontransplant patients with CRKP BSI, ranging from 30 to 50% (38, 39). Of note, in our analysis, BSI was not an independent predictor of death after controlling for severity of illness and therapeutic strategy.

This study had several limitations. First, the data were collected retrospectively; some variables, such as Sequential Organ Failure Assessment scores and the production of carbapenemase, were not included, and some variables, such as medicines, lacked in detail. Second, the underlying mechanisms of resistance were not routinely assessed. We cannot rule out the possibility of emergence of hypervirulent CRKP strains, which has occurred in Chinese hospitals (40, 41). Third, some data reflect the biases at our institution. For example, over the period of the study, a tigecycline-containing regimen was widely used, because CAZ-AVI and polymyxin B were not available in the early phase of the study.

Taken together, we found that in SOTRs, CRKP infection was associated with 38.3% overall 30-day mortality. Independent risk factors for death after CRKP infection included older age, allograft failure, and septic shock, while appropriate targeted therapy was an independent protective factor. Allograft failure was an important risk factor, even after stratifying by the site of infection and the type of organ transplant. Nevertheless, rapid antimicrobial susceptibility testing and appropriate targeted therapy are urgently needed in the transplant population to alleviate the impact of CRKP infections on these SOTRs.

## MATERIALS AND METHODS

### Study population.

Eligible SOTRs who were adults (≥18 years old) admitted to the First Affiliated Hospital of Zhengzhou University between January 2018 and December 2021 with CRKP infections were considered. CRKP infection was defined by any K. pneumoniae on cultures that exhibited *in vitro* nonsusceptibility to any carbapenem. Patients whose medical records lacked sufficient information were excluded. Recurrent infections were excluded; only the first episode of CRKP infection following transplantation was included in our study. This study was approved by the Research Ethics Committee of the First Affiliated Hospital of Zhengzhou University with a waiver of informed consent because of the retrospective nature of the study.

### Data collection.

Data on SOTR were collected retrospectively from the electronic medical records with a standardized report form. Information was collected on the following: demographics (age and gender), comorbidities at the time of CRKP infection (e.g., diabetes, hypertension), laboratory data (neutrophil counts, creatinine, alanine transaminase, total bilirubin, and albumin). For graft characteristics, medications (induction immunosuppression, maintenance immunosuppressants, and antibiotics), transplanted organs, and the date of transplant were collected. Complications that occurred from the diagnosis of CRKP infection to death were recorded and included: length of stay in the ICU, renal replacement therapy, mechanical ventilation, respiratory failure, septic shock, and graft dysfunction. For microbiology, details of the CRKP infection episode (including dates of positive cultures and *in vitro* susceptibilities) were recorded. For outcomes, all-cause 14-day, 30-day, and 6-month mortality were recorded.

### Definition.

CRKP infection was defined by the presence of the microorganism from the blood cultures or other usually sterile body sites together with clinical and/or radiological signs of infection. Carbapenem exposure was defined as any carbapenem administered within 30 days prior to CRKP infection. Appropriate empirical treatment was defined as the use of at least one dose of an antibiotic to which the CRKP organism was susceptible *in vitro* during the first 72 h of infection onset. Appropriate targeted therapy was defined as the use of at least one active antimicrobial agent after the availability of susceptibility test results. Allograft failure was defined as the need for retransplantation of the same organ, return to dialysis for kidney transplant recipients (42), or need for persistent mechanical support in transplant recipients (43, 44). For statistical purposes, terminally ill patients discharged home to die were considered to have died at the time of hospital discharge (45).

### Susceptibility testing of K. pneumoniae isolates.

All isolates identified from study subjects were tested for susceptibility to antibiotics. Strains were identified by the Vitek 2 compact system (bioMérieux, Marcy l’Etoile, France) and the Phoenix100 automated system (Becton, Dickinson, Spark, MD, USA). CRKP refers to isolated K. pneumoniae strains that exhibited *in vitro* nonsusceptibility to at least one of the carbapenems, including meropenem, ertapenem, or imipenem. MICs of aztreonam, ertapenem, imipenem, meropenem, amikacin, gentamicin, polymyxin B, tigecycline, trimethoprim-sulfamethoxazol (TMP-SMX), and ciprofloxacin were tested. The results were interpreted using the Clinical and Laboratory Standards Institute (CLSI) breakpoints. For polymyxin B susceptibility testing, the data were interpreted using the clinical breakpoints published by the European Committee on Antimicrobial Susceptibility Testing (version 6.0). This methodology represents the workflow in the microbiology laboratory at our institution.

### Statistical analysis.

Categorical variables were presented as frequencies and percentages. Numerical variables were summarized as the median (IQR). To investigate the differences in characteristics, management of patients, and clinical outcomes, categorical variables were compared with the χ^2^ test, and continuous variables were analyzed with the Kruskal-Wallis nonparametric test. Kaplan-Meier survival probability estimates were calculated from the date of infection up to 30 days (all-cause mortality), stratified according to therapeutic strategies and infection sites, and assessed with the log-rank test. We assessed the associations between the exposure variable and clinical outcomes using Cox proportional hazards regression models, with the calculation of HRs and 95% CIs, presented in tables, in all patients and by the types of organ transplant and sites of infection. All analyses were done with SPSS software (version 25.0) and GraphPad software (version 9.4.1). A two-sided *P* value of less than 0.05 was considered to indicate statistical significance.
